# Supplementary material for: Trends of in-hospital cardiac arrests in a single tertiary hospital with a mature rapid response system
Source: PLoS One. 2022 Jan 13;17(1):e0262541. doi: 10.1371/journal.pone.0262541 (PMC8757966; doi:10.1371/journal.pone.0262541)
Supplement: S1 Table — (DOCX) [file pone.0262541.s001.docx]

**Supporting information**

**Trends of in-hospital cardiac arrests in a single tertiary hospital with a mature rapid response system**

Hohyung Jung, Ryoung-Eun Ko, Myeong Gyun Ko, Kyeongman Jeon

**S1 Table. Comparison of MET activation, incidence of IHCAs, and in-hospital mortality before and after implementing the automated alert and activation system in August 2016.**

| Variables | Before automated alert and activation system | After automated alert and activation system |
| --- | --- | --- |
| Number of hospitalized patients | 73,308 (72,714–79,476) | 92,526 (64,706–97,086) |
| Number of MET activation  MET dose, /1000 patients | 1,284.0 (1,116.0–1,313.5)  16.4 (15.5–17.4) | 1,530.0 (1,079.0–1,620.0)  16.8 (15.8–17.9) |
| IHCA rate, /1000 patients  P-IHCA rate, /1000 patients  NP-IHCA rate, /1000 patients  I-IHCA rate, /1000 patients | 0.960 (0.946–1.026)  0.192 (0.157–0.200)  0.586 (0.490–0.630)  0.224 (0.167–0.231) | 0.898 (0.736–1.033)  0.124 (0.099–0.167)  0.556 (0.438–0.566)  0.265 (0.174–0.329) |
| In-hospital mortality, /1000 patients | 8.4 (8.1–9.4) | 8.0 (7.6–8.2) |

Values are presented as median (interquartile ranges) or number (%).

MET, medical emergency team; IHCA, in-hospital cardiac arrest; P-IHCA, preventable in-hospital cardiac arrest; NP-IHCA, non-preventable in-hospital cardiac arrest; I-IHCA, inevitable in-hospital cardiac arrest.
